# Endoscopic approaches to the orbit: Transnasal and transorbital, a retrospective case series

**DOI:** 10.1016/j.bas.2024.102770

**Published:** 2024-02-19

**Authors:** Cesare Zoia, Eugenia Maiorano, Sara Borromeo, Giorgio Mantovani, Giannantonio Spena, Fabio Pagella

**Affiliations:** a Neurosurgery Unit, Moriggia Pelascini Hospital, Gravedona e Uniti, Italy; b Department of Surgical Science, University of Pavia, Pavia, Italy; c Department of Otorhinolaryngology, Fondazione I.R.C.C.S. Policlinico San Matteo, Pavia, Italy; d Neurosurgery Unit, Department of Translational Medicine, University of Ferrara, Ferrara, Italy; e Neurosurgery Unit, Fondazione I.R.C.C.S. Policlinico San Matteo, Pavia, Italy

**Keywords:** Orbital surgery, Endoscopy, Transorbital, Transnasal, Skull base

## Abstract

**Introduction:**

Orbital pathologies requiring surgery are traditionally treated by open approach with different techniques depending on the lesion location. Recently, minimally invasive endoscopic approaches, such as the Endoscopic Endonasal Approach (EEA) and the Endoscopic Transorbital Approach (ETA) have been introduced in orbital surgery.

**Research question:**

The purpose of this study is to report the combined experience of the Neurosurgical and Ear-Nose-Throat (ENT) Units in the endoscopic approach of orbital pathologies.

**Material and methods:**

We retrospectively retrieved data on patients treated at our Institution between 2016 and 2021 with endoscopic approach for orbital pathologies. The Clavien-Dindo classification and the Scar Cosmesis Assessment and Rating (SCAR) Scale have been used to assess complications and cosmetic outcomes.

**Results:**

39 patients met the inclusion criteria. EEA (15 patients) or ETA (20 patients) were chosen to approach the lesions. In three cases we used a combination of endoscopic and anterior orbitotomy and in one patient a combination of EEA + ETA. The type of procedure performed was orbital biopsy (9 cases), orbital decompression (6 cases), subtotal resection of the lesion (STR) (8 cases) and total resection of the lesion (GTR) (16 cases). The more frequent postoperative complications were diplopia (5.1%, with 1 case of permanent diplopia), trigeminal paraesthesia and dysesthesia (5.1%), palpebral edema (17.9%), periorbital ecchymosis (7.7%). Mean follow up time was 21 months (range 2–63 months).

**Discussion and conclusion:**

Endoscopic approaches to orbital compartments provide minimally invasive access to every orbital compartment with low complications rate and good cosmetic outcome.

## Highlights

• Endoscopic approach to the orbit provide minimally invasive access to every orbital compartment.
• Most frequent postoperative complications are diplopia, trigeminal paraesthesia, palpebral edema and periorbital ecchymosis.
• 85% of complications are in Clavien-Dindo class I.
• Cosmetic results are overall good, both with endonasal and transorbital approach.
• Combined experience between neurosurgeon and ENT surgeon can provide the best surgical and clinical results.

## 1 Introduction

The orbit is a complex anatomical region that can be affected by a great variability of pathologies.

Traditional approaches to the orbital compartment require open surgery such as anterior orbitotomy, lateral orbitotomy or craniotomy with different types of incision depending on the lesion localization such as pterional, frontoorbitozygomatic approach or Kronlein orbitotomy (Khan and Varvares, 2006), (Hendricks and Cohen-Gadol, 2020). If an osteotomy is required, the procedure is burdened by considerable invasiveness and poor aesthetic outcome yield nevertheless this kind of surgery provides optimal visualization of the surgical field. Endoscopic approaches such as the endonasal (EEA) or the transorbital (ETA) ones are gaining more and more importance in literature and in surgical practice in the last decades as surgical accesses to orbital pathologies (Castelnuovo et al., 2015)– (Signorelli et al., 2015). They are an efficient, safe and minimally invasive way to access the orbit. In this article, we report the experience of the Neurosurgical and ENT Departments of the IRCCS San Matteo, Pavia, Italy in endoscopic surgery for orbital pathologies in every orbital quadrant with a comparison with the case series in the literature.

## 2 Materials and methods

We retrospectively reviewed all the cases of endoscopic assisted surgeries for orbital pathologies performed between 2016 and 2021 in the Neurosurgical and ENT Departments of IRCCS Policlinico San Matteo, Pavia, Italy.

Inclusion criteria were benign or malignant lesions, both primarily intraorbital or intracranial with orbital extension, treated with an endoscopic approach. Orbital lesions treated exclusively with open surgery (orbitotomy or transcranial approaches) were excluded.

All patients underwent evaluation of visual symptoms and ocular motility and the presence of exophthalmos and cranial nerve palsy were analyzed. Specific radiological investigations were performed in all cases (CT and/or MRI) to define the site and the volume of the lesion and its relationship with the muscular cone and with the neighbouring structures.

The orbital pathologies have been treated with ETA or EEA approach depending on the location of the lesion. Combined open and endoscopic approaches were used in a select number of patients.

The surgeries were performed by a multidisciplinary team composed by a ENT surgeon, a Neurosurgeon and, in selected cases, a Maxillofacial surgeon. High-resolution video recording systems (Karl Stortz®) and rigid endoscopes with 0° and 45° lenses (Karl Stortz®) were used. The operation took place under general anaesthesia with the assistance of neuronavigation (Brainlab®).

For the EEA, the patient was placed in supine position with the head fixed on the Mayfield headboard. Tampons soaked in a solution containing Xylometazoline hydrochloride 0.1% + 0.01% of oxybuprocaine hydrochloride were placed in the nostrils and left for 10–15 min to decongest the nasal cavities. Middle turbinate resection, uncinectomy and middle antrostomy were performed to improve the visualization of the operating site. The lamina papyracea was then milled with a high-speed drill. After the incision of the periorbit, removal of the lesion was performed by blunt dissection. In case of decompression due to Basedow's ophthalmopathy, a breach was created by removing a portion of the lamina papyracea to allow orbital fat herniation.

As previously described (Zoia et al., 2018) ETA procedures were performed with patient in supine position with the head fixed on the Mayfield headboard. The skin incision can be made below the eyebrow (in the lateral portion about 4 cm long), at the upper eyelid (about 3 cm long) or at the lower eyelid, depending of lesion location. The orbicularis muscle was incised to visualize the orbital bone margin and then the periorbit was detached. After the gentle displacement of the orbital contents, the endoscope was inserted to proceed with the dissection of the periorbit. Once the lesion has been visualized, its resection was carried out by blunt dissection. In case of dura mater incision, we performed a skull base repair with collagen buffer coated with fibrinogen and thrombin (TachoSil®, Takeda Austria GmbH) and fibrin glue.

After surgery, all patients underwent periodic clinical, radiological and endoscopic follow-up. During the postoperative period, the progression of surgical healing, the onset or persistence of signs and symptoms and the onset of complications or relapse were evaluated using the Clavien-Dindo classification (Clavien et al., 2009) and the Scar Cosmesis Assessment and Rating (SCAR) Scale (Kantor, 2016).

## 3 Results

Between 2016 and 2021, 39 (25 women, M:F = 1:1.78) patients underwent endoscopic surgery for orbital pathologies including primary orbital pathologies and intracranial lesions with orbital extension.

Mean age at the time of surgery was 57.2 years (range 27–78 years). The symptoms were exophthalmos (56.4%; 22 patients), visual deficit (30.7%; 12 patients), impaired ocular motility (17.9%; 7 patients) and cranial nerve deficit (12.8%; 4 patients with trigeminal nerve deficit and 1 patient with facial nerve deficit). Other commonly associated symptoms were eyelid edema (10.3%; 4 patients), ptosis (10.3%; 4 patients) and ocular pain (7.7%; 3 patients).

Treated pathologies are shown in Table 1.

**Table 1:** Treated orbital pathology

Orbital pathology	n	%
**Meningioma**	**11**	**28.2**
**Graves ophthalmopathy**	**6**	**15.4**
**Orbital metastases**	**3**	**7.7**
**Orbital carcinoma**	**3**	**7.7**
**Cavernous hemangioma**	**2**	**5.1**
**Orbital mucocele**	**2**	**5.1**
**Dermoid cyst**	**1**	**2.6**
**Fibromyxoid sarcoma**	**1**	**2.6**
**Orbital Varix**	**1**	**2.6**
**Foreign body**	**1**	**2.6**
**Orbital mastocytosis**	**1**	**2.6**
**Other**	**7**	**17.9**

Demographics, symptoms, location of the pathology, selected approach, definitive diagnosis, procedure performed, post-operative complications and post-operative symptoms are shown in Table 2.

**Table 2:** Patients characteristics

Case n°	Age, Sex	Exop.	Red. Ocular mot.	side	quadrant	Conal localization	Surgical approach	Histological findings	Procedure	Complications	FU symptoms
**1**	71,F	no	yes	left	SM	intra/extra	EEA	Normal histology	biopsy	no	no
**2**	49,M	yes	no	Left	M	intra	EEA	Graves ophtalmopathy	decompression	no	no
**3**	71,M	yes	no	Right	S	extra	EEA	Mucocele	aspiration and drainage	no	no
**4**	27,F	yes	no	right	M	intra	EEA	Graves ophtalmopathy	decompression	Epistaxis	no
**5**	27,F	yes	no	Left	M	intra	EEA	Graves ophtalmopathy	decompression	no	no
**6**	37,M	yes	no	Right	M	intra	EEA	Graves ophtalmopathy	decompression	Diplopia	Diplopia
**7**	76,F	no	no	Left	IM	extra	EEA	Cavernous hemangioma	GTR	no	Trigeminal dyesthesia
**8**	45,F	yes	yes	Right	M	intra	EEA	Cavernous hemangioma	GTR	no	no
**9**	40,M	no	no	Left	M	extra	EEA	Orbital lesion	biopsy	no	no
**10**	73,M	yes	no	Right	M	intra	EEA	Graves ophtalmopathy	decompression	Diplopia	no
**11**	35,F	yes	no	Left	M	intra	EEA	Graves ophtalmopathy	decompression	no	Exophtalmos relapse
**12**	67,F	yes	no	Left	apex	intra	EEA	Meningioma	GTR	Palpebral edema	Relapse
**13**	43,M	No	yes	Bilat	S	extra	EEA	Mucocele	aspiration and drainage	Periorbital abscess	no
**14**	72,F	No	yes	Right	apex	extra	EEA	Adenoidocystic carcinoma	biopsy	no	no
**15**	55,F	No	no	Left	apex	intra	EEA	Cyistic lesion	GTR	no	no
**16**	35,F	No	no	Left	posterior	intra	EEA + open	Dermoid cyst	STR	Right half-face paraesthesia	no
**17**	67,F	Yes	yes	Left	apex	extra	EEA + ETA	Adenoidocystic carcinoma	biopsy	no	no
**18**	71,F	Yes	no	Right	SL	extra	ETA	Meningioma	biopsy	no	no
**19**	36,F	Yes	no	Right	posterior	extra	ETA	Meningioma	STR	Palpebral edema	Abscess
**20**	46,F	No	no	Left	posterior	extra	ETA	Meningioma	STR	Ecchymosis	no
**21**	50,F	Yes	yes	Right	posterior	extra	ETA	Meningioma	GTR	Ecchymosis	no
**22**	72,F	Yes	yes	Left	SL	intra	ETA	Varix	GTR	Partial deficit RL	no
**23**	58,F	No	no	Left	posterior	extra	ETA	Mastocitosis	GTR	no	no
**24**	65,M	No	no	Left	SL	extra	ETA	Meningioma	GTR	no	no
**25**	59,M	No	no	Right	posterior	extra	ETA	Metastasis	GTR	no	Relapse
**26**	79,M	Yes	yes	Left	S	extra	ETA	Poorly diff.carcinoma	STR	no	no
**27**	42,F	No	no	Right	posterior	extra	ETA	Meningioma	GTR	Palpebral edema	no
**28**	48,F	Yes	yes	Right	SL	intra	ETA	Meningioma	STR	Palpebral edema	no
**29**	72,F	No	no	Bilat	posterior	extra	ETA	Normal histology	biopsy	no	no
**30**	74,F	Yes	no	Left	L	extra	ETA	Meningioma	STR	Palpebral edema	no
**31**	69,M	Yes	no	Left	S	intra/extra	ETA	Normal histology	STR	Palpebral edema	Infraorbital n. Dysesthesia
**32**	64,M	No	no	Left	apex	intra	ETA	Inflammatory infiltrate	biopsy	no	no
**33**	77,F	Yes	no	Right	apex	extra	ETA	Meningioma	GTR	Palpebral edema	no
**34**	55,F	No	no	Left	SL	extra	ETA	Fibromyxoid sarcoma	GTR	no	no
**35**	67,M	Yes	yes	Left	SL	intra/extra	ETA	Metastasis	GTR	no	relapse
**36**	42,F	Yes	no	Right	IM	intra/extra	ETA	Metastasis	biopsy	no	Corneal ulcer
**37**	49,F	No	no	Right	posterior	intra	ETA	Foreign body	GTR	no	no
**38**	78,F	Yes	yes	Right	L	extra	ETA + open	Meningioma	STR	no	no
**39**	67,M	No	no	Right	I	extra	ETA + open	Normal histology	biopsy	no	no

EEA = endoscopic endonasala approach ETA = endoscopic transorbital approach Exop. = exophtalmos GTR = gross total resection I = inferiore IM = inferomedial L = lateral M = medial Red.Ocular Mot = reduced ocular motility S = superior SL = superolateral SM = superomedial STR = subtotal resection.

**Fig. 1:**
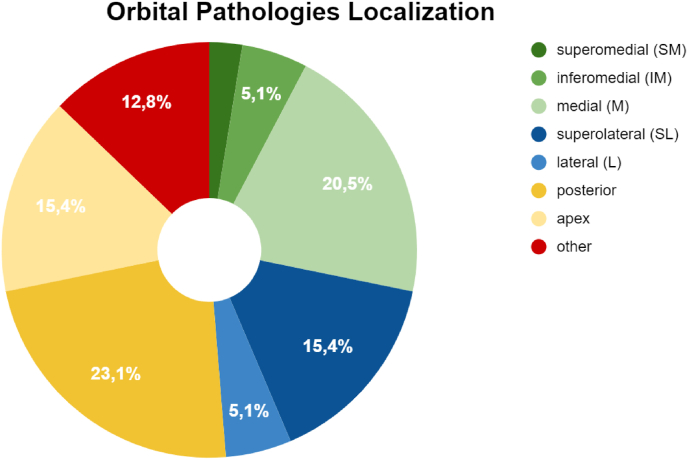
Orbital pathologies localization.

Fig. 1 shows the location of the pathologies.

**Fig. 2:**
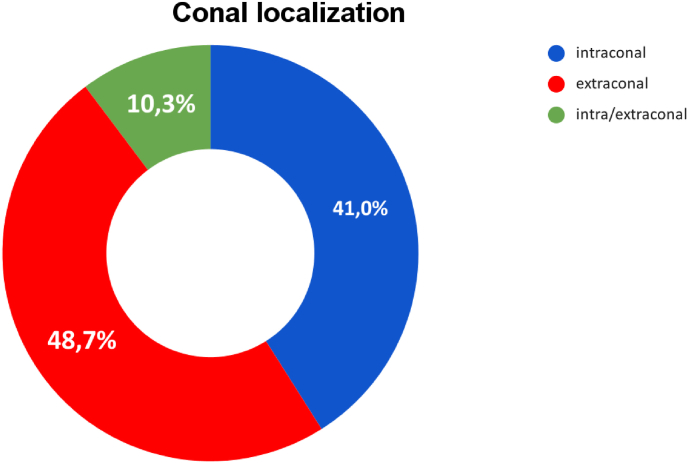
Intra/extra conal lesions classification.

Depending on the relationship with the muscular cone, the pathologies were divided into intraconal or extraconal. Fig. 2 shows the percentage value of the ratio of the lesions to the muscle cone.

The procedures performed were 9 orbital biopsies, 6 orbital decompressions, 8 subtotal resections (STR) of the lesion and 16 gross total resections of the lesion (GTR).

The approaches chosen were transorbital (ETA) in 51.3% of cases (20 patients) and transnasal (EEA) in 38.5% of cases (15 patients). In 3 cases (7.7%) we used endoscopic surgery in association with anterior orbitotomy (in 1 case with incision of the lower eyelid, 1 case with incision of the upper eyelid and 1 case with a supraciliary incision) and in 1 case EEA and ETA were used in combination.

**Fig. 3:**
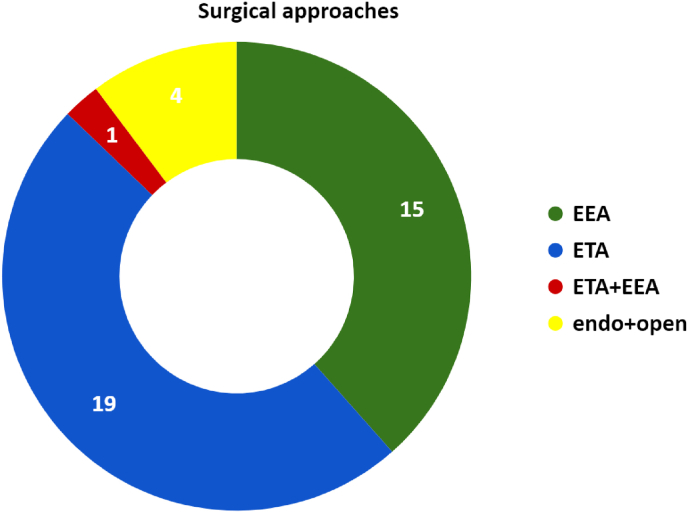
Surgical approaches.

**Fig. 4:**
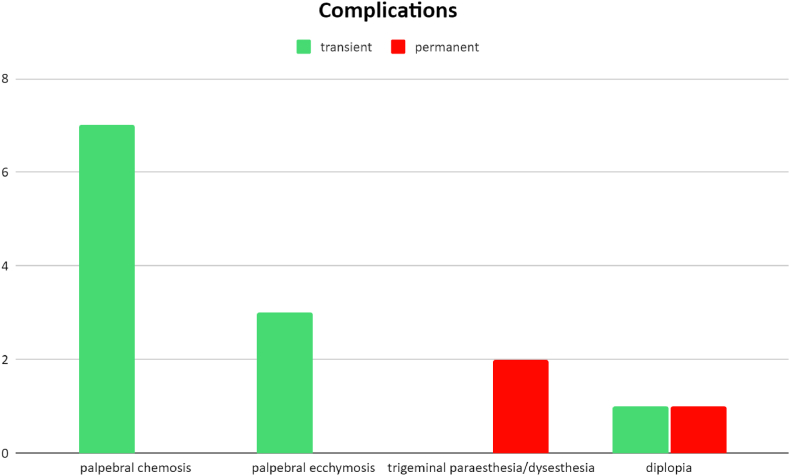
Complications.

Fig. 3 summarizes the types of approaches used. During the postoperative period, in the majority of cases (76.9%) patient’s clinical conditions remained unchanged or improved. The most frequent postoperative complications (all Clavien-Dindo class 1) were diplopia (5.1%, with 1 case of permanent diplopia), eyelid chemosis (17.9%), periorbital ecchymosis (7.7%) and trigeminal paraesthesia/dysesthesia (5.1%) (see Fig. 4).

In one case, after the surgery the patient suffered from major epistaxis which required ligation of the sphenopalatine artery (Clavien-Dindo class 3a).

In two cases, infections developed following the surgical procedure: a periorbital abscess following removal and drainage of mucocele and a brain abscess following removal of meningioma 9 months and 1 month after surgery, respectively (Clavien-Dindo class 3 b).

The main complications have been reported in Fig. 4, making a distinction between transient complications (in green) and permanent ones (in red) and in Table 3.

**Fig. 5:**
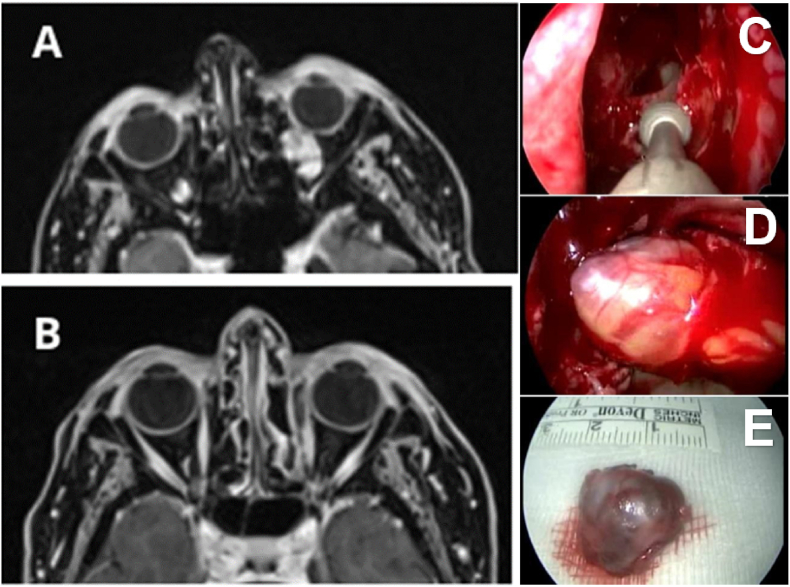
(A) Preoperative MRI showing the presence of a mass in the inferomedial quadrant of the orbit with rich enhancement (B) Postoperative MRI showing total excision of the lesion (C, D) endoscopic endonasal surgery with ethmoidectomy. (E) Operative piece following excision. Source: Archive image IRCCS San Matteo di Pavia ENT department.

**Table 3:** Complications

Complications	transient	%	permanent n °	%	Clavien-Dindo
Diplopia	1	2.56	1	2.56	1
Paraesthesia/dysesthesia V c.n.	0	0	2	5.13	1
Eyelid edema	7	17.95	0	0	1
Ecchymosis	6	15.38	0	0	1
Epistaxis	1	2.56	0	0	3a
Periorbital abscess	1	2.56	0	0	3 b
Brain abscess	1	2.56	0	0	3 b

After performing either a biopsy, subtotal resection (STR) or a gross total resection (GTR), the biological sample was analyzed by the Pathological Anatomy Laboratory at our Institution. The lesions had histological characteristics of malignancy in 48.7% of cases (19 lesions). Histological findings are shown in Table 4. Mean follow up time was 21 months (range 2–63 months).

**Table 4:** Histological findings

Histological findings	n°	%
Meningiotheliomatous meningioma	8	26.67
Metastasis	3	10
Fibroblastic meningioma	2	6.67
Adenoidocystic carcinomas	2	6.67
Cavernous hemangioma	2	6.67
Poorly differentiated carcinoma	1	3.33
Mucocele	1	3.33
Mastocitosis	1	3.33
Fibromixoid sarcoma	1	3.33
Dermoid cyst	1	3.33
Other	7	23.33

Recurrence rate after GTR (13 patients) during the follow up has been 23.07%. Relapses occurred in two cases following resection of metastases (one with pulmonary primitivity and the other with oropharyngeal primitivity) and in one case following removal of a meningioma.

All patients showed a good cosmetic outcome of the surgical scar, reporting 0 at the SCAR scale.

### 3.1 Illustrative cases

Case A Cavernous angioma treated with endoscopic endonasal approach (EEA)

A 76-year-old patient presented with dizziness, left eye pain and frontal headache.

MRI detected a mass of 10 × 10 × 10 mm in the inferomedial quadrant of the left orbit in the extraconal space characterized by an intense contrast enhancement suspicious for cavernous hemangioma (Fig. 5).

The patient underwent EEA and GTR was achieved. During the follow-up (36 months), the patient reported dysesthesia in the trigeminal nerve territory (first branch) that didn’t require therapy. No recurrence or wound healing issues were detected.

Case B Orbital meningioma treated with endoscopic transorbital approach (ETA)

A 77-year-old patient presented with hypovisus and exophthalmos in the right eye. The MRI showed an anterior temporal meningioma with involvement of the sphenoid bone and engagement of the right orbital apex, the size of the intraorbital mass was 29 × 27 × 24 mm. The patient underwent ETA via right upper eyelid incision with subsequent dural closure (two-layers technique).

During the postoperative period, the patient showed improvement in right vision and rapidly resolving eyelid edema.

**Fig. 6:**
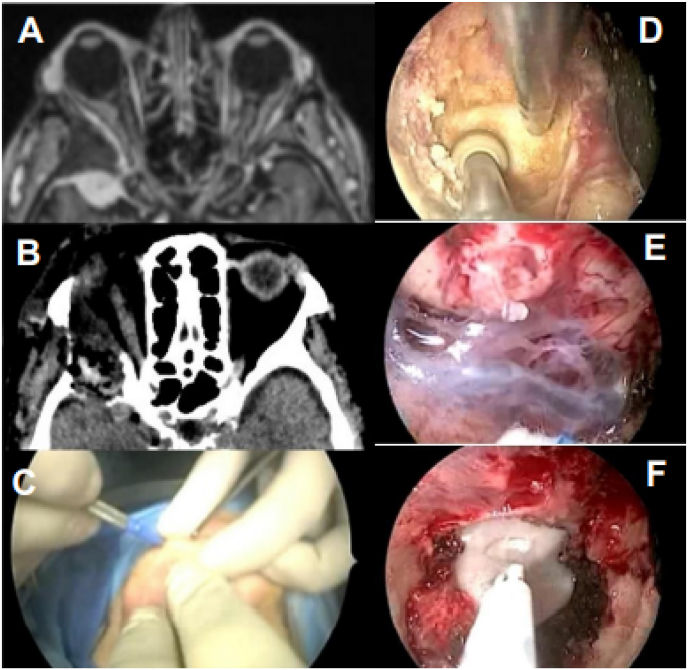
(A) Preoperative MRI showing the presence of temporopolar meningioma with involvement of the sphenoid and of the lateral wall of the orbit (B) postoperative CT showing the orbital decompression and removal of the lesion (C) right upper eyelid incision (D, E) intraoperative endoscopic views (F) dural plastic.

Post-operative CT showed the total resection of the orbital lesion and the decompression of the orbital compartment (Fig. 6).

## 4 Discussion

The choice of the best surgical approach to treat orbital pathologies is determined by many factors: mainly the location of the lesion, its extension, its histological features and the likelihood of damaging the adjacent neurovascular structures. Other important factors to consider are the ease of access to the site and the final cosmetic outcome.

Traditional approaches to orbital lesions involve open surgery with different techniques including anterior orbitotomy, lateral orbitotomy or the zygomatic fronto-temporo-orbital approach (Bejjani et al., 2001). This study is focused on the endoscopic approach to the orbital pathology and how this type of approach can reach every orbital compartment with reduced surgical trauma, better cosmetic results yield with the same clinical outcome.

Over the last few years, endoscopic techniques have been used more frequently to treat both orbital and skull base pathologies with EEA and ETA, in a continuously evolving landscape (Zoia et al., 2023).

As for the choice of the best access to the orbit, several “Round the clock” algorithms have been introduced to help the surgeon. These algorithms allow to choose the optimal access to the lesion based on its location (Paluzzi et al.), (Luzzi et al., 2019). In 2021 Jeon et al. proved how to have a 360° access to the orbital compartment with endoscopic approaches (Jeon et al., 2020).

The lesions in the inferomedial quadrants (IM) are approached with EEA, while those in the superolateral (SL) and inferolateral (IL) quadrants are approached with ETA. To approach superomedial lesions (SM), on the other hand, EEA can be used in combination with ETA. ETA is also used as an access corridor for skull base pathologies (Houlihan et al., 2021).

The EEA was described in 1989 by Mc Donogh et al. (McDonogh and Meiring, 1989) for dacryocystorhinostomy and in 1999 by Herman et al. (1999) for the removal of an orbital cavernoma but only recently it has been used more frequently by surgeons thanks to the refinement of the surgical technique and the redefinition of the anatomy of the region from an endoscopic perspective. Furthermore, it is particularly suited in cases of orbital decompression for the treatment of Graves' disease because it guarantees an excellent visualization of the surgical field and avoids external scars (Metson and Pletcher, 2006).

Recently the trans-eyelid transorbital endoscopic approach has been described by Dallan et al. for access to orbital tumors of the lateral quadrants (Dallan et al., 2016). Moe et al. instead described the minimally invasive neuroendoscopic approach through the transorbital corridor (TONES Transorbital Neuroendoscopic Surgery) for lesions of the anterior skull base (Moe et al., 2010).

As already reported by Jeon et al. the results of endoscopic approaches compared to transcranial ones are comparable in terms of clinical outcome while they are clearly better as regards postoperative complications and cosmetic results (Jeon et al., 2020).

Coherently, in our case series complications such as decreased visual acuity, rhinoliquoral fistulas, enophthalmos or facial nerve palsy have never occurred, while they are associated with open surgery (Okay et al., 2010), (Montano et al., 2018). Furthermore, comparing the rates of diplopia, these are comparable in both open and endoscopic approaches, with a permanent diplopia rate in our series of 2.56% while Montano et al. report a rate of III cranial nerve palsy of 2.9%. It is interesting to note that overall 85% of complications (17/20) were rated in Clavien-Dindo class I (any deviation from the normal postoperative course without need of specific therapy or surgery).

Traditional techniques, therefore, allow better exposure of the surgical field and could guarantee a greater probability of complete resection of the lesion in many cases (Corvino et al., 2022), but they are related to a higher variety and frequency of complications. Furthermore, endoscopic approaches have a significant advantage in case of difficult target site with the avoidance of an osteomtomy otherwise necessary with an open approach.

In particular, the transorbital approach can be useful to replace the transcranial open approaches or those that involve lateral orbitotomy in selected cases for lateral lesions to the optic nerve, while the endonasal approach is optimal to replace the transcarnucular or transconjunctival approaches, especially in the case of lesions located in the orbital apex medial to the optic nerve.

In addition to the morbidity related to the type of approach, orbital surgery is considered risky because it requires careful dissection of the very delicate structures contained in the orbital space and it is also complicated by the lack of reference points that can lead to a loss of orientation in the surgical site. Endoscopic techniques, if carried out by specialized and highly trained surgeons, minimize manipulation and guarantee a magnified visualization of the operating field, with the limit, however, of two-dimensional visualization.

Comparing our case series with the literature regarding endoscopic approaches, it seems to be in line with the present case studies, such as the one reported by Locatelli et al. (2020) or by Zoli et al. (2020), as the most frequent complications are diplopia (due to the manipulation of nerves and muscles of the conal compartment) and periorbital edema (due to the post-operative reactive local inflammation) while liquorrhea in our series has never occurred, possibly demonstrating the efficacy of multilayer reconstruction of the skull base.

The use of intraoperative imaging, neuronavigation and advanced instrumentation can further decrease morbidity by helping the surgeon to identify the lesion quickly, thus reducing the invasiveness of the approach, manipulation and retraction of the orbital structures (Newman, 2021).

The main limitations of this study were the limited number of patients, the inclusion of a wide variety of diseases with different nature and histology, and the retrospective design.

## 5 Conclusions

The transnasal or transorbital endoscopic surgical approaches allow treatment of a wide variety of orbital pathologies with a 360° approach, with the possibility of reaching each compartment of the orbit using these techniques individually or in combination with each other.

Our retrospective case series, in comparison with the literature produced so far, demonstrate how the endoscopic approach is effective in the treatment of orbital pathologies and, moreover, how it is related to less invasiveness and fewer complications than traditional open approaches that involve osteotomies. However, further multicentric studies with prospective design are necessary to confirm this evidence.

## Fundings

This research did not receive any specific grant from funding agencies in the public, commercial, or not-for-profit sectors.

## Contributions

Conceptualization: CZ, FP, GS; Methodology: EM, SB, GS; Formal analysis and investigation:EM, SB; Writing - original draft preparation:EM, SB; Writing - review and editing:GM; Supervision: FP.

## Declaration of competing interest

The authors have no competing interests to declare that are relevant to the content of this article.
